# Impacts of the urinary sodium-to-potassium ratio, sleep efficiency, and conventional risk factors on home hypertension in a general Japanese population

**DOI:** 10.1038/s41440-021-00628-y

**Published:** 2021-02-15

**Authors:** Takumi Hirata, Mana Kogure, Naho Tsuchiya, Ken Miyagawa, Akira Narita, Kotaro Nochioka, Akira Uruno, Taku Obara, Tomohiro Nakamura, Naoki Nakaya, Hirohito Metoki, Masahiro Kikuya, Junichi Sugawara, Shinichi Kuriyama, Ichiro Tsuji, Shigeo Kure, Atsushi Hozawa

**Affiliations:** 1 Tohoku Medical Megabank Organization, Tohoku University, Sendai, Japan; 2 Hokkaido University Faculty of Medicine, Sapporo, Japan; 3 Graduate School of Medicine, Tohoku University, Sendai, Japan; 4 OMRON Healthcare Co., Ltd., Muko, Japan; 5 Tohoku University Hospital, Tohoku University, Sendai, Japan; 6 School of Health and Social Services, Saitama Prefectural University, Koshigaya, Japan; 7 School of Medicine, Tohoku Medical and Pharmaceutical University, Sendai, Japan; 8 Teikyo University School of Medicine, Tokyo, Japan; 9 International Research Institute of Disaster Science, Tohoku University, Sendai, Japan

**Keywords:** Population attributable fraction, Home hypertension, Risk factor, Public health, Japanese

## Abstract

Recently, a high urinary sodium-to-potassium (Na/K) ratio and reduced sleep efficiency, in addition to conventional risk factors (obesity and excess alcohol intake), have been identified as risk factors for hypertension. We estimated the population attributable fraction (PAF) for home hypertension due to these risk factors in a general Japanese population. We conducted a cross-sectional study including 1384 participants (393 men and 991 women) to estimate the odds ratio (OR) and 95% confidence interval (CI) for the presence of any of the conventional risk factors using multivariable logistic regression analyses. The models were adjusted for sex, age, smoking status, and log-transformed average daily steps. We also estimated the OR and 95% CI for the presence of any of the overall risk factors. Furthermore, we calculated the PAF due to these risk factors. The results showed that the prevalence of home hypertension was 39.0% (540/1384). The presence of any of the conventional risk factors, as well as any of the overall risk factors, was significantly associated with an increased prevalence of hypertension (OR 2.80, 95% CI 2.15–3.65; OR 2.50, 95% CI 1.93–3.22, respectively). The PAF for hypertension due to the presence of any of the conventional risk factors and the PAF due to the presence of any of the overall risk factors were 30.2% and 39.0%, respectively. In conclusion, the impact of the overall risk factors, including the urinary Na/K ratio and sleep efficiency, on home hypertension was higher than that of conventional risk factors alone. The management of the urinary Na/K ratio and sleep efficiency as well as conventional risk factors might be important in the management of blood pressure.

## Introduction

Hypertension is the leading cause of cardiovascular diseases; thus, the prevention of hypertension is essential to reduce the burden of cardiovascular diseases [1–3]. Obesity and moderate-to-high alcohol consumption are conventional risk factors for hypertension [4–8]. Accordingly, reductions in body weight and alcohol consumption are important measures to control blood pressure [9–14].

The urinary sodium-to-potassium (Na/K) ratio is also a predictive marker for hypertension [15–17]. In the INTERSALT study, the 24-h urinary Na/K ratio had a stronger relationship with blood pressure than the 24-h urinary sodium (Na) level alone [15]. Additionally, a previous study demonstrated that the urinary Na/K ratio in casual spot urine was highly correlated with the urinary Na/K ratio in 24-h urine samples (the gold standard examination) [18]. This means that the evaluation of the urinary Na/K ratio in casual spot urine can be conveniently performed in clinical settings or at health checkups. Similarly, sleep efficiency has been considered to be a predictor of hypertension. In fact, we have shown that reduced sleep efficiency is associated with the prevalence of hypertension [19]. These markers are expected to be lifestyle indicators related to the development of hypertension.

For the primary prevention of hypertension, the estimated population attributable fraction (PAF) provides meaningful information for public health. However, few studies have reported PAFs for hypertension in Japan due to the presence of several risk factors [20–25]. In particular, no previous studies have reported the PAFs for hypertension due to the urinary Na/K ratio and sleep efficiency. In addition, whether the urinary Na/K ratio and sleep efficiency as well as conventional risk factors contributes to the increase in PAFs for hypertension is unknown. Therefore, we estimated the PAFs for home hypertension due to the urinary Na/K ratio, sleep efficiency and conventional risk factors in a Japanese general population.

## Methods

### Participants

This cross-sectional study was conducted to calculate the PAFs for home hypertension due to combinations of risk factors. Participants fulfilling the following criteria were included: (1) completed the follow-up survey in the Tohoku Medical Megabank project Cohort Study (TMM Cohort Study), which is population based and has been ongoing since 2013; (2) were aged ≥20 years and lived in Miyagi Prefecture during the baseline survey from May 2013 to March 2016; (3) visited one community support center (located in Tagajo City in Miyagi Prefecture); and (4) measured home blood pressure, sleep efficiency, and the urinary Na/K ratio and had counted their steps daily for at least 7 days. The participants in this present study were recruited from June 2017 to March 2018. Written informed consent was obtained from each participant. The study was approved by the Institutional Review Board of the TMM Organization (approval number: 2017-4-007).

A total of 1474 participants were initially included. However, 90 participants were excluded for missing data on (1) treatment status for hypertension (*n* = 33); (2) height, body weight, and alcohol consumption status (*n* = 53); and (3) sex, age, and smoking status (*n* = 4). Finally, we analyzed the data of 1384 participants (393 men and 991 women).

### Measurements

1. Measurements of home hypertension
  Home hypertension, the outcome of the present study, was defined as having a morning home blood pressure ≥135/85 mmHg or receiving treatment for hypertension [26]. Information on treatment for hypertension was collected using a self-reported questionnaire. Home blood pressure was measured using a cuff-oscillometric device (HEM-7080IC; Omron Healthcare Co., Ltd., Kyoto, Japan) and was recorded for 10 days. Measurement was performed with the participant in a sitting position after at least 5 min of rest in the morning, within 1 h after awakening. The arm cuff was maintained at heart level during the measurement, and if applicable, the measurements were taken before the participants took their medications for hypertension and ate breakfast and after urination. We defined morning home blood pressure as the average of the first measurements performed each morning.
2. Measurements of conventional risk factors
  We defined obesity and moderate-to-heavy daily alcohol intake as conventional risk factors in the present study. Obesity was defined as body mass index (BMI) ≥ 25 kg/m^2^ based on the Western Pacific Region of the World Health Organization (WHO) criteria for the Japanese population [27]. Height and weight were measured in participants while they were wearing light clothing. BMI was calculated as weight (kg) divided by the square of height (m). Alcohol consumption status was assessed using a self-reported questionnaire, and we divided the participants into three groups (nondrinkers, past drinkers, and current drinkers). In addition, we asked all the current drinkers about the typical quantities of alcohol consumed per occasion and the typical consumption frequency per week for each type of alcohol. We calculated the mean daily alcohol intake as ethanol intake per day, and then, we defined moderate-to-heavy daily alcohol intake as ≥46 g of ethanol intake per day.
3. Measurements of other risk factors
  We defined a high urinary Na/K ratio and reduced sleep efficiency as new risk factors in the present study. The urinary Na/K ratio was measured twice daily (in the morning after awakening and at night before going to bed) using a hand-held urinary Na/K ratio monitor (HEU-001F; Omron Healthcare Co., Ltd., Kyoto, Japan) for 10 continuous days. We used the average of the values for all the measurements in the analysis. All the participants were grouped into four quartiles, and the highest quartile had a ratio ≥5.20. Sleep efficiency was measured using a contactless biomotion sleep sensor (HSL-102M; Omron Healthcare Co., Ltd., Kyoto, Japan) for 10 days. The details of the measurement conducted using the sleep sensor are described elsewhere [28–30]. Sleep efficiency was defined as the ratio of the sleep duration (min) to the time spent in bed (min) multiplied by 100 [31]. We used the average of all measurements in the analysis. We defined reduced sleep efficiency as a sleep efficiency of <85% in the present study [32, 33].
4. Measurements for the covariates
  All the participants completed a self-reported questionnaire to obtain information on sex, age, and smoking status. The daily steps, one of the covariates in the present study, were counted using an activity monitor (HJA-750C; Omron Healthcare Co., Ltd., Kyoto, Japan). The activity monitor was positioned on the waist of each participant and was attached throughout the day, except while the participant was bathing or sleeping, for 11 continuous days. The data collected on the first of the 11 days were excluded. In addition, we excluded the data if the daily total measurement duration was less than 8 h. We used the average of all measurements for this analysis.

### Statistical analysis

Data are presented as the means (standard deviations) or medians (interquartile ranges) for continuous variables or numbers (percentages) for categorical variables. We used the unpaired *t*-test or Wilcoxon rank-sum test for continuous variables and the chi-square test or Fisher’s exact test for categorical variables to compare the characteristics between the presence and absence of conventional or other risk factors. We divided all participants into four groups according to the presence or absence of conventional and other risk factors. Multivariable logistic regression models were used to obtain odds ratios (ORs) and 95% confidence intervals (CIs) to assess the relationship of each conventional and other risk factor with the prevalence of home hypertension. Models were adjusted for sex, age, smoking status, and log-transformed average daily steps. In addition, we calculated the PAF for each group using the following formula: (the proportion of participants with home hypertension)*(Adjusted OR-1)/(Adjusted OR) [7, 34]. Next, we used the multivariable logistic regression model to assess the relationship between the presence of conventional risk factors and the prevalence of hypertension. We also used the multivariable logistic regression model to assess the relationship between the presence of conventional risk factors and prevalent hypertension and the relationship between the presence of conventional and/or other risk factors and prevalent hypertension. Additionally, we performed the same analyses described above stratified by age (<65 years and ≥65 years). Two-sided *P* values of <0.05 were considered statistically significant. All analyses were performed using STATA SE 13 data analysis and statistical software (Stata Corp LP, College Station, TX, USA).

## Results

The clinical characteristics of the participants at the baseline survey according to the type of risk factors are shown in Table 1. Of 1384 participants, 198 (14.3%) had conventional and other risk factors (i.e., highest quartile of urinary Na/K ratio and reduced sleep efficiency), 257 (18.6%) had conventional risk factors only, 280 (20.2%) had other risk factors only, and 649 (46.9%) had neither conventional nor other risk factors. The proportions of participants according to the presence or absence of conventional and/or other risk factors are summarized in Supplemental Table 1. Of 1384 participants, 540 (39.0%) had home hypertension, and the prevalence was highest (64.7%) in participants with conventional and other risk factors. The group with conventional and other risk factors had more men (52.0%) and current smokers (10.1%) and the lowest daily average steps (5.5 × 10^3^ steps per day) compared to the remaining three groups.

**Table 1:** Clinical characteristics of participants according to the combinations of conventional and other risk factors

	Total participants	Conventional (+)	Conventional (+)	Conventional (-)	Conventional (-)
		Other (+)	Other (-)	Other (+)	Other (-)
Number of participants	1384	198	257	280	649
Sex, men	393 (28.4%)	103 (52.0%)	96 (37.4%)	75 (26.8%)	119 (18.3%)
Age, years	58.8 (12.9)	58.3 (13.7)	60.0 (12.0)	55.7 (14.0)	59.9 (12.3)
≥65 years	589 (42.6%)	83 (41.9%)	117 (45.5%)	99 (35.4%)	290 (44.7%)
Current smoker, yes	89 (6.4%)	20 (10.1%)	20 (7.8%)	25 (8.9%)	24 (3.7%)
Average daily steps, 1000 steps per day	6.0 (4.6–7.8)	5.5 (4.2–7.5)	6.1 (4.5–7.8)	6.1 (4.7–8.1)	6.2 (4.7–7.9)
Conventional risk factors for hypertension, yes	455 (32.9%)	198 (100.0%)	257 (100.0%)	0 (0.0%)	0 (0.0%)
Body mass index, kg/m^2^	22.9 (3.3)	26.1 (3.7)	25.6 (3.4)	21.5 (2.0)	21.4 (2.0)
≥25, yes	312 (22.5%)	136 (68.7%)	176 (68.5%)	0 (0.0%)	0 (0.0%)
Current alcohol drinker, yes	673 (48.6%)	141(71.2%)	170 (66.2%)	124 (44.3%)	238 (36.7%)
≥46 g/day, yes	196 (14.2%)	86 (43.4%)	110 (42.8%)	0 (0.0%)	0 (0.0%)
Other risk factors for hypertension, yes	478 (34.5%)	198 (100.0%)	0 (0.0%)	280 (100.0%)	0 (0.0%)
Urinary Na/K ratio	4.4 (1.6)	5.7 (1.7)	3.8 (0.9)	5.9 (1.8)	3.5 (0.9)
≥5.2 (Highest quartile), yes	345 (24.9%)	136 (68.7%)	0 (0.0%)	209 (74.6%)	0 (0.0%)
Sleep efficiency, %	91.1 (6.2)	86.1 (8.2)	92.4 (3.7)	88.2 (7.8)	93.4 (3.5)
<85%, yes	190 (13.7%)	89 (45.0%)	0 (0.0%)	101 (36.1%)	0 (0.0%)
Hypertension, yes	540 (39.0%)	128 (64.7%)	126 (49.0%)	97 (34.6%)	189 (29.1%)
Systolic blood pressure, mmHg	125.0 (16.1)	133.1 (15.3)	129.0 (15.2)	124.4 (16.9)	121.2 (15.1)
Diastolic blood pressure, mmHg	75.0 (9.8)	80.4 (9.7)	77.4 (9.0)	74.7 (9.5)	72.6 (9.3)
Received treatment for hypertension, yes	281 (20.3%)	70 (35.4%)	66 (25.7%)	43 (15.4%)	102 (15.7%)

*Na/K* sodium-to-potassium.

The associations between the presence of each of risk factors, including conventional and/or other risk factors, and home hypertension are shown in Table 2. In multivariate analyses, the presence of each of risk factors was significantly associated with an increased prevalence of hypertension when adjusted for potential confounders (obesity, OR 3.14, 95% CI 2.33–4.25; moderate-to-high alcohol consumption, OR 1.52, 95% CI 1.06–2.19; highest quartile of urinary Na/K ratio, OR 1.40; 95% CI 1.04–1.88; reduced sleep efficiency, OR 2.51, 95% CI 1.71–3.70). The estimated PAFs for hypertension due to obesity, moderate-to-high alcohol consumption, the highest quartile of the urinary Na/K ratio, and reduced sleep efficiency were 23.7%, 6.5%, 7.9%, and 11.6%, respectively.

**Table 2:** Association between each risk factor and home hypertension

	Number of the total participants	Number of participants with home hypertension	OR	95% CI	*P* value	PAF
Conventional risk factors for hypertension						
Body mass index, kg/m^2^						
≥25, yes	312	188	3.14	2.33–4.25	<0.001	23.7%
Current alcohol drinker						
≥46 g/day	196	102	1.52	1.06–2.19	0.024	6.5%
Other risk factors for hypertension						
Urinary Na/K ratio						
≥5.2 (Highest quartile)	345	150	1.40	1.04–1.88	0.025	7.9%
Sleep efficiency, %						
<85%	190	104	2.51	1.71–3.70	<0.001	11.6%
Sex, men			1.80	1.35–2.41	<0.001	
Age, per 10 years			2.14	1.89–2.43	<0.001	
Current smoker, yes			1.49	0.90–2.47	0.125	
Average daily steps, log-transformed			0.87	0.65–1.15	0.319	

*Na/K* sodium-to-potassium, *OR* odds ratio, *CI* confidence interval, *PAF* population attributable fraction.

The association between conventional risk factors and home hypertension and the association between overall risk factors, including conventional and/or other risk factors, and home hypertension are shown in Table 3. In multivariate analyses, the presence of any of conventional risk factors was significantly associated with an increased prevalence of hypertension after adjusting for potential confounders (OR 2.80, 95% CI 2.15–3.65; as shown in Table 3A). The estimated PAF for hypertension in participants with any of the conventional risk factors was 30.2%. On the other hand, the presence of any of the overall risk factors was significantly associated with an increased prevalence of hypertension in multivariate analyses (OR 2.50, 95% CI 1.93–3.22; as shown in Table 3B). The estimated PAF of hypertension in participants with any of the overall risk factors was 39.0%.

**Table 3:** Association between conventional risk factors only or all risk factors and home hypertension

	Number of the total participants	Number of participants with home hypertension	OR	95% CI	*P* value	PAF
(A) Measured only conventional risk factors for hypertension						
Any of conventional risk factors, yes	455	254	2.80	2.15–3.65	<0.001	30.2%
Sex, men			1.99	1.50–2.63	<0.001	
Age, per 10 years			2.00	1.78–2.26	<0.001	
Current smoker, yes			1.56	0.95–2.57	0.081	
Average daily steps, log-transformed			0.81	0.61–1.07	0.142	
(B) Measured all risk factors including conventional risk factors, urinary Na/K ratio and sleep efficiency for hypertension						
Any of all risk factors, yes	735	351	2.50	1.93–3.22	<0.001	39.0%
Sex, men			2.03	1.54–2.68	<0.001	
Age, per 10 years			2.04	1.81–2.29	<0.001	
Current smoker, yes			1.46	0.89–2.41	0.137	
Average daily steps, log-transformed			0.79	0.60–1.05	0.102	

*OR* odds ratio, *CI* confidence interval, *PAF* population attributable fraction, *Na/K* sodium-to-potassium.

The association between conventional risk factors and home hypertension and the association between conventional and/or other risk factors and home hypertension stratified by age are shown in Table 4. In participants aged <65 and ≥65 years, the presence of any of conventional risk factors was significantly associated with an increased prevalence of hypertension after adjusting for potential confounders (<65 years, OR 3.38, 95% CI 2.34–4.87; ≥65 years, OR 2.28, 95% CI 1.55–3.34). The estimated PAFs for hypertension in participants with any of the conventional risk factors aged <65 and ≥65 years were 30.2% and 24.1%, respectively. Similarly, in these age groups, the presence of any of conventional and/or other risk factors was significantly associated with an increased prevalence of hypertension in multivariate analyses (<65 years, OR 2.70, 95% CI 1.85–3.93; ≥65 years, OR 2.35, 95% CI 1.65–3.34). The estimated PAFs for hypertension among participants with any of conventional and/or other risk factors aged <65 and ≥65 years were 44.9% and 35.2%, respectively.

**Table 4:** Association between conventional risk factors only or all risk factors and home hypertension stratified by age

	Number of the total participants	Number of participants with home hypertension	OR	95% CI	*P* value	PAF
(A) Measured only conventional risk factors for hypertension						
(A-1) <65 years old	795	202				
Any of conventional risk factors, yes	255	109	3.38	2.34–4.87	<0.001	38.0%
Sex, men			2.71	1.74–4.21	<0.001	
Age, per 10 years			2.13	1.74–2.60	<0.001	
Current smoker, yes			1.40	0.77–2.55	0.273	
Average daily steps, log-transformed			0.86	0.55–1.33	0.494	
(A-2) ≥65 years old	589	338				
Any of conventional risk factors, yes	200	145	2.28	1.55–3.34	<0.001	24.1%
Sex, men			1.70	1.18–2.47	0.005	
Age, per 10 years			1.30	0.83–2.03	0.255	
Current smoker, yes			1.68	0.62–4.56	0.308	
Average daily steps, log-transformed			0.73	0.51–1.06	0.096	
(B) Measured all risk factors including conventional risk factors, urinary Na/K ratio and sleep efficiency for hypertension						
(B-1) <65 years old	795	202				
Any of all risk factors, yes	436	144	2.70	1.85–3.93	<0.001	44.9%
Sex, men			2.89	1.87–4.46	<0.001	
Age, per 10 years			2.18	1.79–2.66	<0.001	
Current smoker, yes			1.35	0.74–2.44	0.325	
Average daily steps, log-transformed			0.81	0.52–1.24	0.334	
(B-2) ≥65 years old	589	338				
Any of all risk factors, yes	299	207	2.35	1.65–3.34	<0.001	35.2%
Sex, men			1.67	1.15–2.42	0.007	
Age, per 10 years			1.32	0.84–2.07	0.228	
Current smoker, yes			1.49	0.55–4.03	0.435	
Average daily steps, log-transformed			0.73	0.51–1.06	0.098	

*OR* odds ratio, *CI* confidence interval, *PAF* population attributable fraction, *Na/K* sodium-to-potassium.

## Discussion

The results of the present study showed that both conventional and other risk factors were significantly positively related to the prevalence of home hypertension. The PAF for hypertension due to any of the conventional risk factors was 30.2%, and that due to any of the conventional and/or other risk factors increased to 39.0%. In age-stratified analyses, both the PAF for hypertension due to any of conventional risk factors and that due to any of conventional and/or other risk factors in participants aged <65 years were higher than those in participants aged ≥65 years.

All established risk factors, including obesity, moderate-to-high alcohol consumption, the highest quartile of the urinary Na/K ratio, and reduced sleep efficiency, were also significantly associated with home hypertension in the present study. We found the largest PAFs to be due to obesity, followed by reduced sleep efficiency, the highest quartile of the urinary Na/K ratio, and moderate-to-high alcohol consumption. Previous studies have shown that the PAFs for hypertension due to obesity or BMI were the largest among several risk factors for hypertension [20, 21]. Our findings are consistent with those of these previous studies, which suggests that the reduction in body weight should be a priority for the prevention of hypertension in the context of global public health. In fact, a meta-analysis of randomized controlled trials (RCTs) revealed that weight reduction of 5.1 kg decreased systolic blood pressure by 4.78 mmHg and diastolic blood pressure by 3.56 mmHg [9]. Additionally, reduced alcohol consumption and sodium intake and increased potassium intake had favorable effects on the prevention of hypertension in meta-analyses of RCTs [35–37]. These findings suggest that these are modifiable risk factors that are worth managing to prevent hypertension.

In the present study, the PAF for hypertension due to the presence of any of conventional risk factors was 30.2%, and the PAF due to the presence of any of conventional and/or other risk factors increased to 39.0%. The magnitude of the OR of the presence of any of conventional risk factors only is higher than that of the presence of any of the overall risk factors, and the increased PAF due to the presence of any of the overall risk factors merely reflects the increased prevalence of participants with risk factors. However, in participants without conventional risk factors, the presence of the highest quartile of urinary Na/K ratio or reduced sleep efficiency was significantly associated with the prevalence of hypertension (OR, 1.68; 95% CI, 1.19–2.37). Therefore, the urinary Na/K ratio and sleep efficiency contributed to the maintenance of blood pressure even in individuals without conventional risk factors. As mentioned earlier, the favorable effect of decreased sodium intake and increased potassium intake on blood pressure was clarified in previous intervention studies [36, 37]. On the other hand, although some interventional approaches, such as sleep hygiene education and cognitive behavioral insomnia therapy, improve sleep efficiency [38, 39], the effect on blood pressure reduction has not been sufficiently clarified [40, 41]. Further studies are warranted to explore the effect of interventional approaches to improve sleep efficiency on the prevention of hypertension.

Regardless of the age group, the PAF for hypertension due to conventional and/or other risk factors was higher than that due to conventional risk factors in the present study. Moreover, both PAF due to conventional risk factors and PAF due to conventional and/or other risk factors among participants aged <65 years were higher than those among participants aged ≥65 years. The frequency of risk was almost the same between the two age groups in the present study; however, the magnitude of the risk among participants aged <65 years was higher than that among participants aged ≥65 years. Previous studies have also shown that the risk of developing hypertension among younger individuals was higher than that among older individuals [23]. These risk factors increase the activity of the sympathetic nervous system, which leads to increased blood pressure [42, 43], and the activity of the sympathetic nervous system decreases with age [44]. Accordingly, our findings suggest that the management of all risk factors, including conventional risk factors, the urinary Na/K ratio and sleep efficiency, is important, particularly in younger patients, to reduce the burden of hypertension.

The strengths of the present study included the measurement of the urinary Na/K ratio and sleep efficiency using simple and objective devices, adopting a self-monitoring and self-control approach that could be widely used in the general population. In addition, the present study focused on home hypertension, not office hypertension. Previous studies have reported that home blood pressure is superior to office blood pressure for predicting left ventricular hypertrophy and the risk of future cardiovascular events [45, 46]. However, we need to consider the limitations when interpreting the results of this study. First, this was a cross-sectional study; thus, the causal relationships between risk factors and hypertension could not be established. Further studies are warranted to estimate PAFs due to the risk factors in longitudinal studies. Second, the PAF is usually calculated based on the risk ratio in cohort studies. We used the PAF in this cross-sectional study because the concept of PAF is important to indicate the importance of reducing risk factors in the field of public health, and the PAF was used for this purpose in the previous cross-sectional study [7]. The value of PAF in the present study should be interpreted with caution because the prevalence of hypertension was not low. Third, we set the highest quartile (i.e., 5.20) as the cutoff value for the risk of hypertension in the present study, but the association between the urinary Na/K ratio and the prevalence of hypertension was linear, and it is difficult to determine the cutoff value of the urinary Na/K ratio for the risk of hypertension. The validity of the cutoff value needs to be verified in the future.

In conclusion, the urinary Na/K ratio and/or sleep efficiency increased the burden of hypertension in the present study. This finding was shown regardless of the age group. The management of the urinary Na/K ratio and sleep efficiency as well as conventional risk factors is desired to prevent hypertension.

## Supplementary information

Supplementary Table 1
