# Supplementary material for: Impacts of the urinary sodium-to-potassium ratio, sleep efficiency, and conventional risk factors on home hypertension in a general Japanese population
Source: Hypertens Res. 2021 Feb 15;44(7):858–65. doi: 10.1038/s41440-021-00628-y (PMC8255210; doi:10.1038/s41440-021-00628-y)
Supplement: Supplementary file 1 — Supplementary Table 1 [file 41440_2021_628_MOESM1_ESM.docx]

Supplementary Table 1: Proportion of participants based on the presence or absence of conventional and/or other risk factors

|  |  | Conventional risk factors | | | | Total number of participants |
| --- | --- | --- | --- | --- | --- | --- |
|  |  | Obesity and moderate-to-high current drinkers | Obesity only | Moderate-to-high current drinkers only | No conventional risk factor |  |
|  |  |  |  |  |  |  |
| Other  risk  factors | Highest quartiles of urinary Na/K ratio and reduced sleep efficiency | 3 (0.2%) | 15 (1.1%) | 9 (0.7%) | 30 (2.2%) | 57 (4.1%) |
|  | Highest quartiles of urinary Na/K ratio only | 14 (1.0%) | 56 (4.0%) | 39 (2.8%) | 179 (12.9%) | 288 (20.8%) |
|  | Reduced sleep efficiency only | 7 (0.5%) | 41 (3.0%) | 14 (1.0%) | 71 (5.1%) | 133 (9.6%) |
|  | No other risk factor | 29 (2.1%) | 147 (10.6%) | 81 (5.9%) | 649 (46.9%) | 906 (65.5%) |
| Total number of participants | | 53 (3.8%) | 259 (18.7%) | 143 (10.3%) | 929 (67.1%) | 1,384 (100.0%) |

Abbreviation: Na/K, sodium-to-potassium
